# LncRNA kcnq1ot1 promotes lipid accumulation and accelerates atherosclerosis via functioning as a ceRNA through the miR-452-3p/HDAC3/ABCA1 axis

**DOI:** 10.1038/s41419-020-03263-6

**Published:** 2020-12-09

**Authors:** Xiao-Hua Yu, Wen-Yi Deng, Jiao-Jiao Chen, Xiao-Dan Xu, Xian-Xia Liu, Lei Chen, Meng-Wen Shi, Qi-Xian Liu, Min Tao, Kun Ren

**Affiliations:** 1 Institute of Clinical Medicine, The Second Affiliated Hospital of Hainan Medical University, Haikou, 570100 Hainan PR China; 2 Department of Pathology, The First Affiliated Hospital of Anhui Medical University, Hefei, 230032 Anhui PR China; 3 Department of Cardiology, The Second Affiliated Hospital of Hainan Medical University, Haikou, 570100 Hainan PR China; 4 The First School of Clinical Medicine, Anhui Medical University, Hefei, 230032 Anhui PR China; 5 Department of Pathophysiology, School of Basic Medical Sciences, Anhui Medical University, Hefei, 230032 Anhui PR China

**Keywords:** Long non-coding RNAs, Dyslipidaemias

## Abstract

Kcnq1 overlapping transcript 1 (kcnq1ot1), an imprinted antisense lncRNA in the kcnq1 locus, acts as a potential contributor to cardiovascular disease, but its role in atherosclerosis remains unknown. The aim of this study was to explore the effects of kcnq1ot1 on atherogenesis and the underlying mechanism. Our results showed that kcnq1ot1 expression was significantly increased in mouse aorta with atherosclerosis and lipid-loaded macrophages. Lentivirus-mediated kcnq1ot1 overexpression markedly increased atherosclerotic plaque area and decreased plasma HDL-C levels and RCT efficiency in apoE^−/−^ mice fed a Western diet. Upregulation of kcnq1ot1 also reduced the expression of miR-452-3p and ABCA1 but increased HDAC3 levels in mouse aorta and THP-1 macrophages. Accordingly, kcnq1ot1 overexpression inhibited cholesterol efflux and promoted lipid accumulation in THP-1 macrophages. In contrast, kcnq1ot1 knockdown protected against atherosclerosis in apoE^−/−^ mice and suppressed lipid accumulation in THP-1 macrophages. Mechanistically, kcnq1ot1 enhanced HDAC3 expression by competitively binding to miR-452-3p, thereby inhibiting ABCA1 expression and subsequent cholesterol efflux. Taken together, these findings suggest that kcnq1ot1 promotes macrophage lipid accumulation and accelerates the development of atherosclerosis through the miR-452-3p/HDAC3/ABCA1 pathway.

## Introduction

Atherosclerosis, which is characterized by excessive lipid deposition within the arterial intima, is the pathological basis of most cardiovascular disease, such as myocardial infarction and stroke^1^. During atherogenesis, circulating monocytes transmigrate into the subintima and then differentiate into macrophages that uptake large amounts of modified lipoprotein to form foam cells, a hallmark of early-stage atherosclerotic lesions^2^. ATP binding cassette transporter A1 (ABCA1) is a transmembrane protein that mediates the efflux of free cholesterol (FC) to apolipoprotein A-I (apoA-I) to produce nascent high-density lipoprotein (HDL) particles. ABCA1-mediated cholesterol efflux is regarded as the first and rate-limiting step of reverse cholesterol transport (RCT), a process in which excessive peripheral cholesterol is transported by HDL to the liver for excretion into the bile and feces^3^. Previous studies from our groups and others have demonstrated that fargesin^4^, pemafibrate^5^ and xanthohumol^6^ promote RCT and reduce atherosclerotic plaque area by upregulating ABCA1 expression in apolipoprotein E-deficient (apoE^−/−^) mice. Therefore, a better understanding of the molecular mechanisms underlying ABCA1 regulation is of critical importance to develop novel therapeutic strategies for atherosclerosis.

Long non-coding RNAs (lncRNAs), a class of transcripts with lengths exceeding 200 nucleotides, play an important role in the regulation of gene expression by affecting chromatin modification, transcription, or post-transcriptional processing^7^. There is accumulating evidence that aberrant expression of lncRNAs is associated with lipid metabolism disorder and atherosclerosis^8–10^. Kcnq1 overlapping transcript 1 (kcnq1ot1), which is mapped to chromosome 11p15.5 in human beings, is an imprinted antisense lncRNA in the kcnq1 locus^11^. Increased kcnq1ot1 levels are observed in peripheral blood monocyte cells isolated from patients with coronary artery disease^12^. Importantly, silencing of kcnq1ot1 has been shown to mitigate myocardial ischemia/reperfusion injury^13^. It is still unclear, however, whether kcnq1ot1 is involved in the development of atherosclerosis.

Histone acetylation can stimulate gene transcription by loosening interaction between DNA and histones. This is a reversible process involving histone acetyltransferases (HATs) and histone deacetylases (HDACs)^14^. Among these enzymes, myeloid HDAC3 deletion was shown to increase ABCA1 expression in macrophages and promote plaque stability in a mouse model of atherosclerosis^15^. MicroRNA-452-3p (miR-452-3p) is located in human chromosome Xq28 and correlates with metabolic diseases^16–18^. A large number of lncRNAs can bind to specific miRNAs to competitively increase target gene expression, called competing endogenous RNAs (ceRNAs)^19^. A recent study showed that kcnq1ot1 functions as a ceRNA to induce cardiac fibroblast pyroptosis in diabetic cardiomyopathy^20^. However, whether kcnq1ot1 sponges miR-452-3p to regulate HDAC3 and ABCA1 expressions needs to be addressed.

In this study, we found that kcnq1ot1 dramatically reduces RCT and aggravates atherosclerosis in apoE^−/−^ mice. Importantly, kcnq1ot1 increases the levels of HDAC3 by acting as a ceRNA for miR-452-3p, thereby inhibiting ABCA1 expression and subsequent cholesterol efflux from THP-1 macrophages. Thus, targeting kcnq1ot1 may be a promising therapeutic strategy for the prevention and treatment of atherosclerotic cardiovascular disease.

## Materials and methods

### Mice and diet

All experiments were conducted according to protocols approved by the Animal Care and Use Committee at the Second Affiliated Hospital of Hainan Medical University. Ninety male 8-week-old apoE^−/−^ mice on a C57BL/6 background and 10 wild-type C57BL/6 mice were obtained from Changzhou Cavens Lab Animal Co., Ltd (Jiangsu, China), and housed under a 12 h light/dark cycle with free access to drinking water and food in an environmentally controlled room (24 ± 2 °C, 60% humidity). Among these, ten wild-type C57BL/6 mice and apoE^−/−^ mice were fed a normal chow diet and a Western diet containing 21% fat and 0.3% cholesterol, respectively. After 12 weeks, all mice were sacrificed and the aorta was extracted to detect kcnq1ot1 expression. The remaining 80 apoE^−/−^ mice were fed a Western diet. Among these, 60 mice were randomized into three groups (*n* = 20 per group): control group, lentiviral empty vector (LV-NC) group and lentiviral vector expressing kcnq1ot1 (LV-kcnq1ot1, Shanghai Genechem Co., Ltd, Shanghai, China) group. When the Western diet started, mice were injected with PBS, LV-NC or LV-kcnq1ot1 (2 × 10^9^ TU/mL) via the tail vein once every three weeks. During this process, mice were weighed once every two weeks. Other 20 mice were injected with 2 × 10^9^ TU/mL of lentiviral vectors expressing short hairpin RNA (shRNA) targeting kcnq1ot1 (LV-shkcnq1ot1, Shanghai Genechem Co., *n* = 10) or scrambled control shRNA (LV-shNC, *n* = 10) via the tail vein once every three weeks. After 12 weeks, mice were sacrificed to collect the heart, aorta and blood. Sodium pentobarbital anesthesia was conducted throughout all surgeries. For the isolation of mouse peritoneal macrophages (MPMs), mice were intraperitoneally injected with 4% thioglycollate broth and flushed with 5 mL of sterile PBS. Following centrifugation at 300 rpm for 5 min at 4 °C, cells were collected, counted, and resuspended in RPMI 1640 medium (Gibco, Grand Island, NE, USA) supplemented with 10 % fetal bovine serum (FBS).

### Assessment of En face lesion area

After euthanasia, the whole aorta was dissected from apoE^−/−^ mice, and the adventitial tissues were carefully removed. The aorta was unfolded longitudinally, stained with Oil Red O, and then photographed. The percentage of lesion area stained by Oil Red O in the aortic surface was determined by Image-Pro Plus 7.0 software.

### Analyses of atherosclerotic lesions in the aortic root

Mice were exsanguinated under anesthesia, and the upper portion of the heart and proximal aorta were separated carefully. After washing with PBS, they were embedded in Optimal Cutting Temperature compound (O.C.T, Sakura Finetek Japan Co., Ltd, Tokyo, Japan) and stored at −20 °C. Serial 8-μm-thick cryosections throughout the three aortic valves were collected using a cryostat microtome and placed on glass slides. Oil Red O, hematoxylin-eosin (HE) and Masson staining were then performed. Image-Pro Plus 7.0 software was used for quantitative analyses.

### Detection of serum lipid levels

Blood samples were obtained from the retro-orbital plexus of apoE^−/−^ mice. Plasma levels of total cholesterol (TC), triglycerides (TG), HDL-cholesterol (HDL-C) and low-density lipoprotein cholesterol (LDL-C) were measured by enzymatic methods using commercial kits (Nanjing Jiancheng Biotech Inc, Jiangsu, China).

### In vivo RCT assay

J774 macrophages were incubated with 50 μg/mL acetylated LDL (Yiyuan biotechnology, Guangzhou, China) and 5 μCi/mL [^3^H]-cholesterol (PerkinElmer, MA, USA) for 48 h. Subsequently, the cells were washed, equilibrated and resuspended in ice-cold Dulbecco’s modified Eagle’s medium (DMEM, Gibco). The labeled cells were intraperitoneally injected into apoE^−/−^ mice (4.5 × 10^6^ cells/mouse, *n* = 5 per group). Plasma samples were collected via saphenous vein puncture at 6, 24, and 48 h after injection, and radioactivity in 10 μL aliquots was measured using a liquid scintillation counter. The feces were continuously collected until 48 h, vacuum dried and homogenized in 50% NaOH overnight. Then, 20 µl aliquots were used for scintillation counting. At the end of the study, the mice were sacrificed to isolate the liver. The hepatic tissue (100 mg) was washed in ice-cold PBS, mixed with hexane/isopropanol (3:2) for 48 h and then dried overnight for lipid extraction and radioactivity detection. The RCT efficiency was calculated as the ratio of radioactivity in the plasma, liver, or feces to total radioactivity injected at baseline.

### Cell culture and lentiviral vector transfection

Human THP-1 monocytes were purchased from American Type Culture Collection (ATCC, USA). Cells were cultured in RPMI 1640 medium containing 1% penicillin–streptomycin (Beyotime, Shanghai, China) and 10% FBS in a humidified atmosphere of 5% CO_2_ at 37 °C. Then, 100 nM phorbol 12-myristate 13-acetate (PMA, Sigma-Aldrich, St. Louis, MO, USA) was added to induce the differentiation of monocytes into macrophages. THP-1 macrophages were transduced with LV-NC, LV-kcnq1ot1, LV-shNC or LV-shkcnq1ot1 at a multiplicity of infection of 100 in the presence of 8 mg/mL of polybrene. Cells in control group were treated with PBS only. After 72 h of incubation, cells were maintained in fresh complete medium (10% FBS + RPMI). qRT-PCR was performed to determine transfection efficiency.

### miR-452-3p mimic/inhibitor transfection and HDAC3 knockdown

THP-1 macrophages were transfected with 50 nM of miR-452-3p mimic/inhibitor or their negative controls (GenePharma, Shanghai, China) using Lipofectamine 3000 reagent (Invitrogen, CA, USA) for 48 h according to the manufacturer’s protocol. Then, qRT-PCR was conducted to assess the transfection efficiency. Both HDAC3 small interfering RNA (siRNA) and scrambled siRNA were designed and synthesized by Ribobio (Guangzhou, China). The siRNAs (80 nM) were transfected into THP-1 macrophages using Lipofectamine 3000 reagent for 24 h. Western blot was performed to observe the efficiency of HDAC3 knockdown.

### Bioinformatics prediction and luciferase reporter assay

The online databases including TargetScan (http://www.targetscan.org/), miRDB (http://mirdb.org/miRDB/) and DIANA-LncBase v.2 (http://carolina.imis.athena-innovation.gr/diana_tools/web/index.php?r=lncbasev2/index) were used to predict the interaction between miR-452-3p and HDAC3 or kcnq1ot1. 293T cells were seeded in a 96-well plate at a density of 15,000 cells per well. The sequences of kcnq1ot1 and HDAC3 3′UTR containing the putative miR-452-3p binding site were amplified by PCR from THP-1 macrophages and then inserted into the downstream of pmirGLO plasmids (Promega, WI, USA), designated as kcnq1ot1-WT and HDAC3-WT. The putative miR-452-3p binding site was mutated to generate corresponding mutant plasmids (kcnq1ot1-Mut and HDAC3-Mut). The 293T cells were co-transfected with the above constructs and miR-452-3p mimic using Lipofectamine 3000 reagent for 48 h. The firefly and renilla luciferase activities were assessed using the commercial Dual-Luciferase reporter assay system (Promega) on a microplate luminometer, and the firefly luciferase activity was normalized to the renilla luciferase activity.

### Pull-down assay with biotinylated miR-452-3p (bio-miR-452-3p)

The pull-down assay was performed as described previously^21^. Briefly, THP-1 macrophages were transfected with bio-miR-452-3p-WT, bio-miR-452-3p-Mut or negative control (bio-NC) for 48 h. Cells were washed with PBS and then lysed on ice for 10 min. The lysates were incubated with M-280 streptavidin magnetic beads (Sigma-Aldrich) at 4 °C for 3 h. To block nonspecific binding of RNA and protein complexes, the beads were coated with RNase-free BSA and yeast tRNA (Sigma-Aldrich). Afterwards, the beads were washed twice with the lysis buffer, three times with low-salt buffer, and once with high-salt buffer. Following isolation by using TRIzol reagent (Invitrogen), the bound RNAs were subjected to qRT-PCR to detect kcnq1ot1 expression.

### RNA isolation and qRT-PCR

Total RNA was extracted from the obtained tissues and cultured cells using TRIzol reagent (Invitrogen). The purity and concentration of the extracted RNA was evaluated using a Nanodrop 3000 (ThermoFisher, Scotts Valley, CA, USA). A high-capacity cDNA reverse transcription kit (Takara, Kyoto, Japan) was used to synthesize complementary DNA. Then, qRT-PCR was performed using SYBR® Premix Ex TaqTM II reagent kit (Takara) on a ABI 7900HT Fast Real-Time PCR System (Applied Biosystems, Foster City, CA, USA) for 45 cycles (95 °C for 3 min, 95 °C for 15 s, and 60 °C for 1 min). U6 was used as internal control for miR-452-3p and β-actin for all others. The primers used are listed in Supplementary Table 1, which were synthesized by Shanghai Sangon Biotech Co., Ltd (Shanghai, China). The specificity of all PCR products was assessed by melting curve analysis. Relative gene expression was analyzed using the 2^−ΔΔ^Ct method.

### Western blot analysis

The tissues and cultured cells were lysed by RIPA buffer (Beyotime) containing 0.1 mmol/L phenylmethylsulfonyl fluoride. Protein extracts were quantified and then subjected to SDS-PAGE, followed by immunoblotting with mouse monoclonal antibody against ABCA1 (ab18180, 1:500, Abcam, Cambridge, MA, USA), rabbit monoclonal antibody against ABCG1 (ab52617, 1:1000, Abcam), rabbit monoclonal antibody against CD36 (ab133625, 1:500, Abcam), mouse polyclonal antibody against SR-A (AF1797, 1:1000, R&D Systems, MN, USA), rabbit monoclonal antibody against LXRα (ab176323, 1:500, Abcam), rabbit polyclonal antibody against HDAC1 (ab53091, 1:1000, Abcam), rabbit monoclonal antibody against HDAC3 (ab32369, 1:1000, Abcam), mouse monoclonal antibody against HDAC5 (ab50001, 1:300, Abcam), mouse monoclonal antibody against HAT-1 (sc-390562, 1:500, Santa Cruz, TX, USA), or rabbit monoclonal antibody against β-actin (ab115777, 1:1000, Abcam). After rinsing with PBS-T, the membranes were incubated with HRP-labeled secondary antibodies (1:3000, Beyotime). The proteins were visualized using Tanon 5500 (Shanghai, China) and BeyoECL Plus (Beyotime), and β-actin was used as an internal control.

### Cholesterol efflux assay

The cholesterol efflux experiments were performed according to our previous method^22^. Briefly, THP-1 macrophages and MPMs isolated from apoE^−/−^ mice were incubated with 50 µg/mL ox-LDL and 0.2 µCi/mL [^3^H]-cholesterol for 48 h. Then, cells were washed with PBS and maintained in RPMI 1640 medium containing 0.1% BSA overnight to equilibrate [^3^H]-cholesterol. After washing with PBS, cells were cultured in 2 mL of RPMI 1640 medium containing 0.5% BSA and 25 μg/mL apoA-I (Sigma-Aldrich) or 50 μg/mL HDL (Sigma-Aldrich) overnight. Radioactivity of [^3^H]-cholesterol in the medium and cells was measured using a liquid scintillation counter, respectively. The percent cholesterol efflux was calculated as the ratio of radioactivity in the medium to total radioactivity (medium + cells).

### High-performance liquid chromatography (HPLC)

Intracellular lipid contents were detected by HPLC as previously described^23^. After treatment, THP-1 macrophages were washed three times and broken by an ultrasonic processor (Scientz, Zhejiang, China) on ice. The protein concentration of cell lysates was measured using a BCA Protein Assay Kit (Beyotime). The cell lysates were then suspended and vortexed. Cholesterol was extracted with n-hexane-isopropanol (3:2, V/V), dissolved in isopropanol (50 mg/mL), and stored at −20 °C. Cholesterol standard calibration solution ranging from 0 to 50 mg/mL was prepared. The reaction mixture, which was composed of MgCl_2_ (500 mM), Tris-HCl (500 mM, pH = 7.4), dithiothreitol (10 mM) and 5% NaCl, was added to 100 μL of cholesterol standard calibration solution and cell solution. Subsequently, 0.4 U cholesterol oxidase was supplemented to detect FC content, and 0.4 U cholesterol oxidase combined with 0.4 U cholesterol esterase was supplemented for measurement of TC content. After incubating at 37 °C for 30 min, the reaction was terminated, and the supernatant was collected. Absorbance at 216 nm was detected. Data were analyzed using TotalChrom software (PerkinElmer).

### Assessment of intracellular lipid droplets by Oil red O staining

THP-1 macrophages were fixed in 4% paraformaldehyde for 8 min and washed three times in PBS. These cells were stained with prepared Oil Red O working solution in the dark at 37 °C for 5 min, and then destained with 60% isopropanol for 10 s. Images of positively stained cells (red) were taken by a fluorescent microscope (Olympus BX50) at ×400 magnification.

### Statistical analysis

All data are presented as the mean ± standard deviation (SD). The cellular experiments were repeated 3–5 times, and the animal experiments were replicated 5–20 times. A two-tailed Student’s *t*-test was used to compare the differences between two groups. One-way ANOVA or two-way ANOVA was used to compare the differences among multiple groups. Statistical analyses were performed using GraphPad Prism 8.0 software (CA, USA). A *P* value less than 0.05 was considered statistically significant.

## Results

### Kcnq1ot1 promotes atherosclerotic plaque formation in apoE^−/−^ mice

**Fig. 1:**
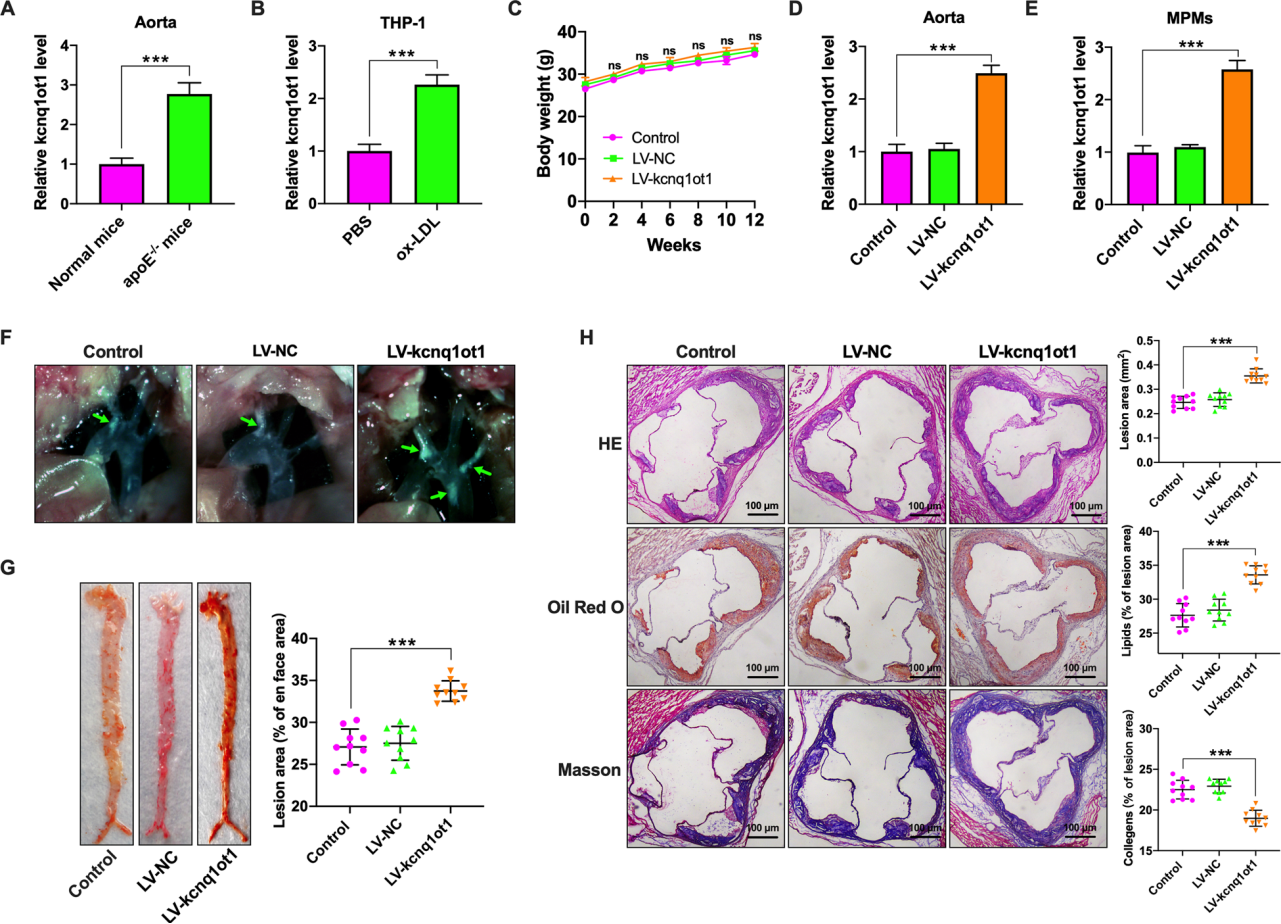
Kcnq1ot1 aggravates atherosclerosis in apoE^−/−^ mice. **A** C57BL/6 mice and apoE^−/−^ mice were fed a normal chow diet and a Western diet for 12 weeks, respectively (*n* = 10 per group). Kcnq1ot1 expression in the aorta was detected by qRT-PCR. B THP-1 macrophages were treated with PBS or 50 µg/mL ox-LDL for 48 h, followed by qRT-PCR analysis of kcnq1ot1 expression (*n* = 3). **C**–**H** Western diet-fed apoE^−/−^ mice were injected via the tail vein with PBS, LV-NC, or LV-kcnq1ot1 (*n* = 20 in each group). **C** Comparison of weight. **D**, **E** The qRT-PCR analysis of kcnq1ot1 expression in the aorta and MPMs. **F** The plaques (green arrows) in the aortic arch of apoE^−/−^ mice under a stereoscopic microscope. **G** The entire aorta was stained with Oil Red O and the atherosclerotic lesion area was quantified by analyzing Oil Red O-positive region on *en face* preparations (*n* = 5 per group). **H** Sections of the aortic root were stained with HE, Oil Red O, or Masson. Lesion area and percentage was quantified using Image-Pro Plus 7.0 software (*n* = 10 per group). Scale bar = 100 μm. Data are represented as mean ± SD. ***P < 0.001; ns not significant vs. control group.

To determine whether kcnq1ot1 expression is altered during atherogenesis, we first detected its expression in the aortic tissues using qRT-PCR. As shown in Fig. 1A, kcnq1ot1 expression was higher in apoE^−/−^ mice fed a Western diet than that in wild-type mice fed a normal chow diet. Consistently, the levels of kcnq1ot1 were significantly increased in ox-LDL-treated THP-1 macrophages compared with unstimulated cells (Fig. 1B). These findings suggest that kcnq1ot1 may play a role in the development of atherosclerosis.

To validate this finding, apoE^−/−^ mice were injected with PBS, LV-NC or LV-kcnq1ot1 and fed a Western diet for 12 weeks. Mice in three groups had a similar weight gain over the course of the study (Fig. 1C). At the end of the study, over 2-fold elevation of kcnq1ot1 levels was observed in the aorta and MPMs of apoE^−/−^ mice transduced with LV-kcnq1ot1 (Fig. 1D, E). Moreover, kcnq1ot1 overexpression increased the number and size of atherosclerotic lesions in the aortic arch regions (Fig. 1F) and *en face* aorta (Fig. 1G). HE, Oil Red O, and Masson staining of cross-sections of the aortic root showed that kcnq1ot1 overexpression also markedly raised lesion area, promoted lipid deposition, and decreased collagen content (Fig. 1H). On the other hand, transduction with LV-shkcnq1ot1 led to an ~76% reduction of kcnq1ot1 expression in the aorta isolated from apoE^−/−^ mice compared with LV-shNC group (Supplementary Fig. 1A). Knockdown of kcnq1ot1 decreased lesion area, inhibited lipid deposition, and increased collagen content, as evidenced by HE, Oil Red O and Masson staining (Supplementary Fig. 1B). Collectively, these in vivo results indicate that kcnq1ot1 functions as an important contributor to atherosclerosis.

### Kcnq1ot1 decreases plasma HDL-C levels and inhibits RCT in apoE^−/−^ mice

**Fig. 2:**
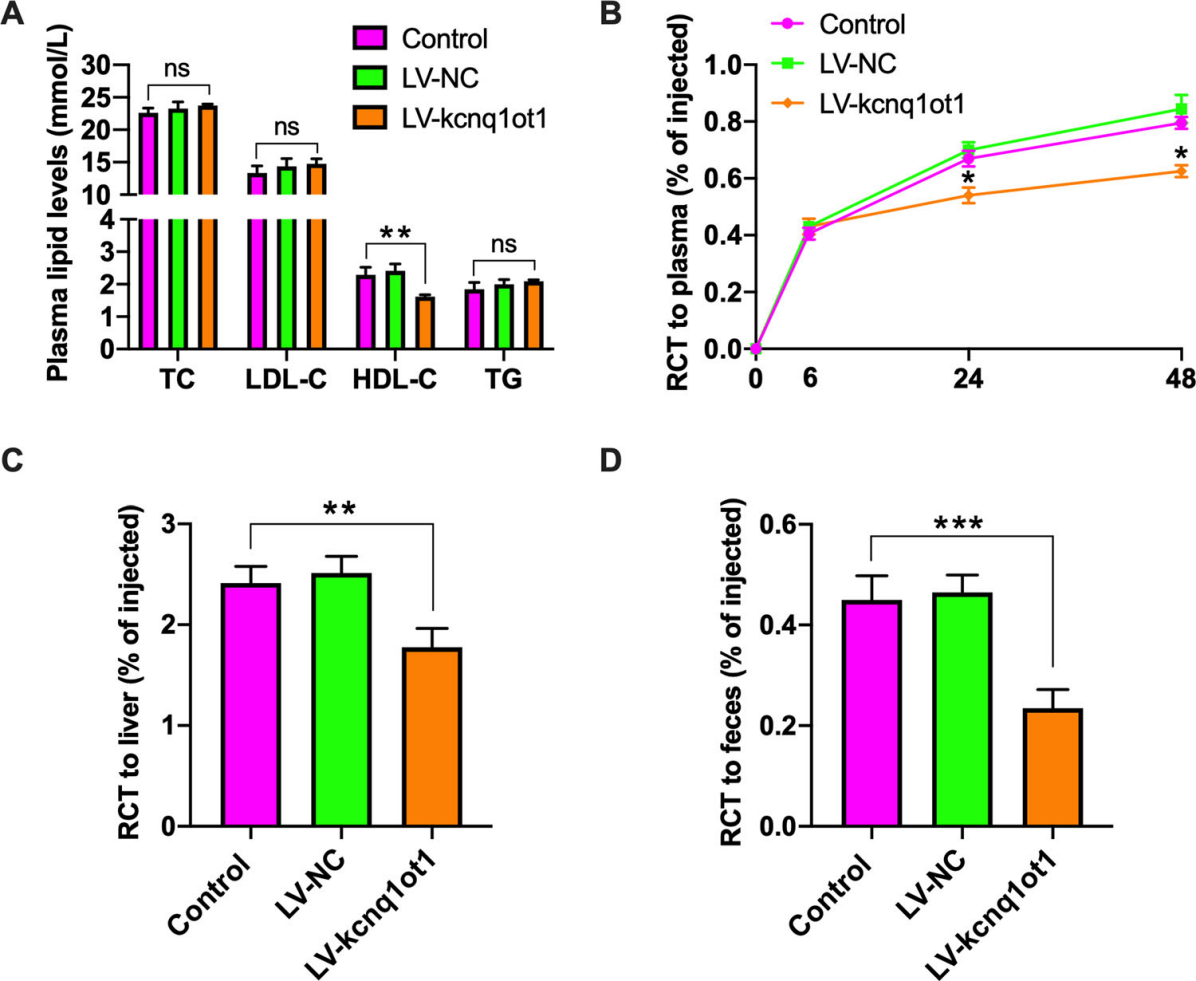
Effects of kcnq1ot1 on plasma HDL-C levels and RCT in apoE^−/−^ mice. **A** Plasma levels of TC, TG, HDL-C, and LDL-C were assessed by enzymatic methods (*n* = 10 per group). **B**–**D** J774 macrophages loaded with [^3^H]-cholesterol and ox-LDL were injected into the abdominal cavity of apoE^−/−^ mice (*n* = 5 per group). The radioactivity in the plasma, liver and feces were measured by a liquid scintillation counter. Data are represented as mean ± SD. *P < 0.05, **P < 0.01, ***P < 0.001; ns not significant vs. control group.

Given lipid metabolism disorder as an independent risk factor of atherosclerosis, the enzymatic methods was used to detect plasma lipid profile. Figure 2A shows that kcnq1ot1 overexpression dramatically decreased plasma HDL-C levels. However, plasma levels of TC, LDL-C and TG trended higher in LV-kcnq1ot1 group compared to other two groups but were not significantly different (Fig. 2A). RCT is regarded as a major pathway to eliminate excessive cholesterol from the body. We then examined the effect of kcnq1ot1 on RCT. Our results showed that apoE^−/−^ mice injected with LV-kcnq1ot1 displayed a significant reduction of [^3^H]-cholesterol content in the plasma, liver, and feces (Fig. 2B–D), suggesting an inhibitory effect of kcnq1ot1 on RCT.

### Kcnq1ot1 inhibits cholesterol efflux from macrophages and promotes intracellular lipid accumulation

**Fig. 3:**
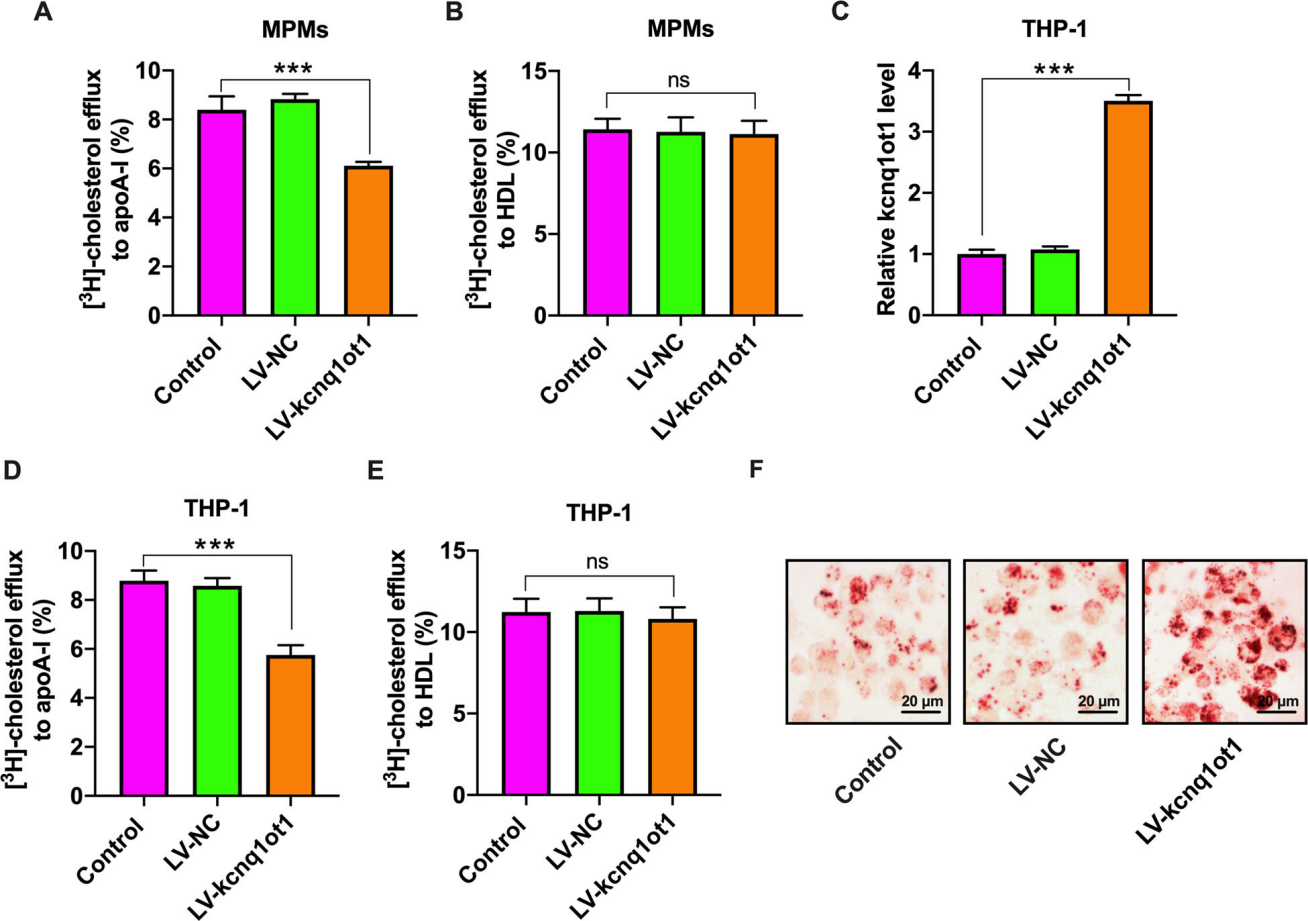
Effects of kcnq1ot1 on cholesterol efflux and lipid accumulation in MPMs and THP-1 macrophages. **A**, **B** After 48 h of incubation with [^3^H]-cholesterol, liquid scintillation counter was used to measure cholesterol efflux from MPMs to apoA-I or HDL (*n* = 5). **C**–**F** THP-1 macrophages were pretreated with or without 50 µg/mL ox-LDL for 48 h, and then transduced with PBS, LV-NC, or LV-kcnq1ot1 for 72 h (*n* = 3). **C** kcnq1ot1 expression was detected by qRT-PCR. **D**, **E** The efflux of cholesterol to apoA-I or HDL. **F** Representative images of Oil red O staining (400×). Scale bar = 20 μm. Data are represented as mean ± SD. ***P < 0.001; ns not significant.

Cholesterol efflux is the first and rate-limiting step of RCT. We isolated MPMs from apoE^−/−^ mice and found that kcnq1ot1 overexpression reduced cholesterol efflux to apoA-I, but not HDL (Fig. 3A, B). To further confirm these effects, we treated THP-1 macrophages with LV-kcnq1ot1 or LV-shkcnq1ot1 to overexpress (Fig. 3C) or silence (Supplementary Fig. 2A) kcnq1ot1. Consistent with data obtained from MPMs, kcnq1ot1 overexpression also suppressed cholesterol transport from THP-1 macrophages to apoA-I but did not affect cholesterol export to HDL (Fig. 3D, E). Accordingly, THP-1 macrophages stably overexpressing kcnq1ot1 exhibited a significant increase in TC, FC, and CE amounts (Supplementary Table 2). In addition, lipid droplets were significantly increased in response to LV-kcnq1ot1, as shown by Oil Red O staining (Fig. 3F). Conversely, silencing of kcnq1ot1 dramatically diminished intracellular TC, FC, and CE levels (Supplementary Table 3). Lipid droplets were fewer in LV-shkcnq1ot1 group compared with LV-shNC group (Supplementary Fig. 2B). These observations suggest that impaired cholesterol efflux from macrophages plays a central role in kcnq1ot1-aggravated lipid accumulation and atherosclerosis.

### Kcnq1ot1 downregulates ABCA1 expression in vivo and in vitro

**Fig. 4:**
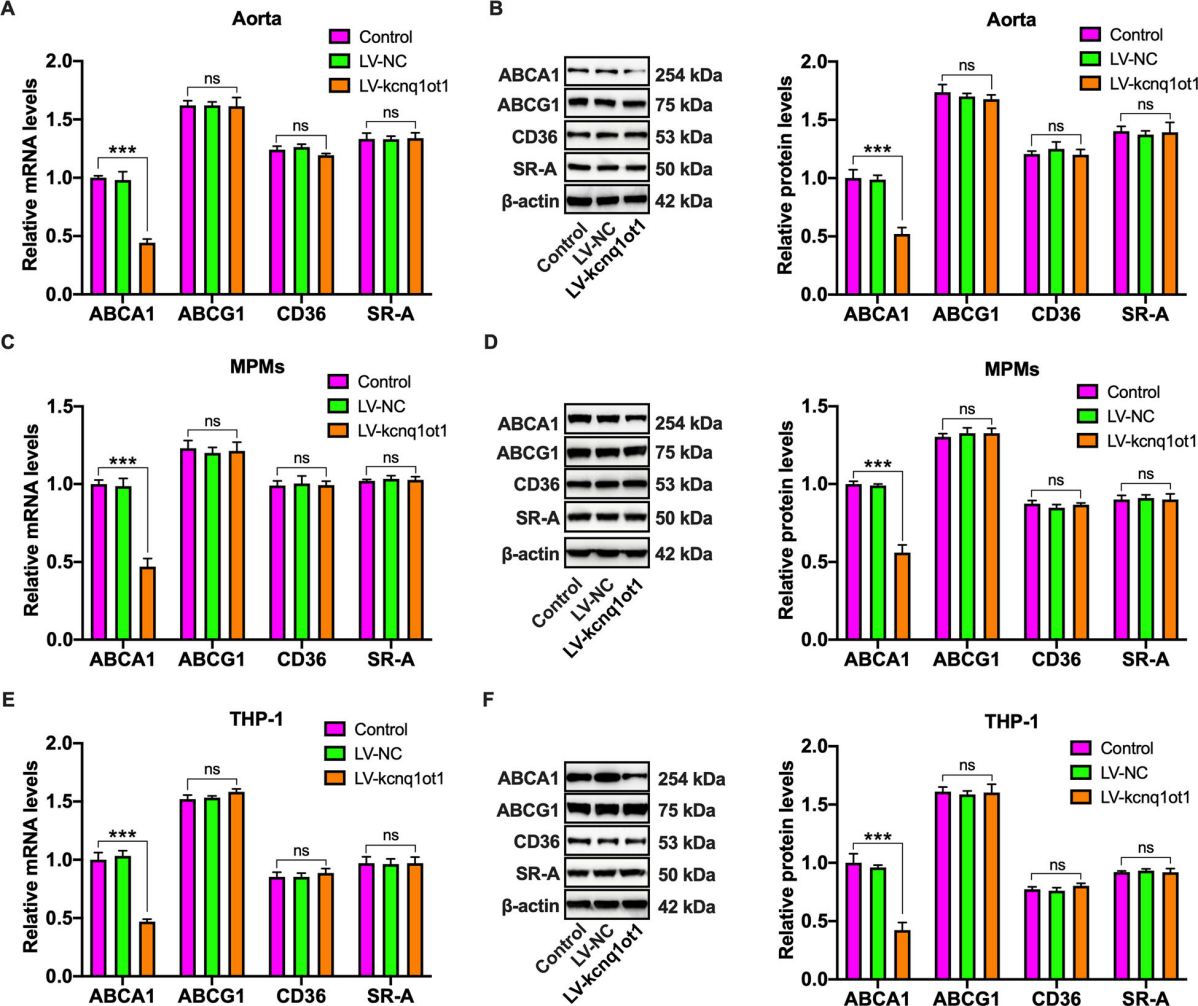
Effects of kcnq1ot1 on cholesterol transporter and receptor expression. A, B The mRNA and protein levels of ABCA1, ABCG1, CD36, and SR-A in the aorta from apoE^−/−^ mice were assayed by qRT-PCR and western blot, respectively (*n* = 10). **C**, **D** qRT-PCR and western blot analyses of ABCA1, ABCG1, CD36 and SR-A expression in MPMs from apoE^−/−^ mice (*n* = 5). **E**, **F** After treatment of THP-1 macrophages with PBS, LV-NC or LV-kcnq1ot1 for 72 h, both qRT-PCR and western blot were employed for detection of ABCA1, ABCG1, CD36, and SR-A expressions. Data are represented as mean ± SD. ***P < 0.001; ns not significant.

It is well known that ABCA1 mediates initial transport of FC to apoA-I for producing nascent HDL particles, while ABCG1 facilitates subsequent continued FC efflux to these HDL particles for further maturation^24^. The above studies have demonstrated that kcnq1ot1 has a detrimental effect on cholesterol efflux from macrophages to apoA-I. To gain insights into potential mechanisms, we next assessed the effects of kcnq1ot1 on ABCA1 and ABCG1 expressions by qRT-PCR and western blot. As expected, kcnq1ot1 overexpression decreased the mRNA and protein levels of ABCA1 but not ABCG1 in the aorta and MPMs (Fig. 4A–D). A similar result was also obtained in THP-1 macrophages overexpressing kcnq1ot1 (Fig. 4E, F), suggesting that kcnq1ot1 inhibits cholesterol efflux by downregulating ABCA1 expression. In addition to decreased cholesterol efflux, foam cell formation is associated with increased cholesterol influx^25^. To exclude the possibility that macrophage lipid accumulation is due to increased cholesterol influx, we detected the expression of CD36 and SR-A, the major receptors responsible for ox-LDL uptake by macrophages. As illustrated in Fig. 4A–F, kcnq1ot1 overexpression did not alter the mRNA and protein levels of these two receptors.

### HDAC3 is involved in kcnq1ot1-induced downregulation of ABCA1 expression

**Fig. 5:**
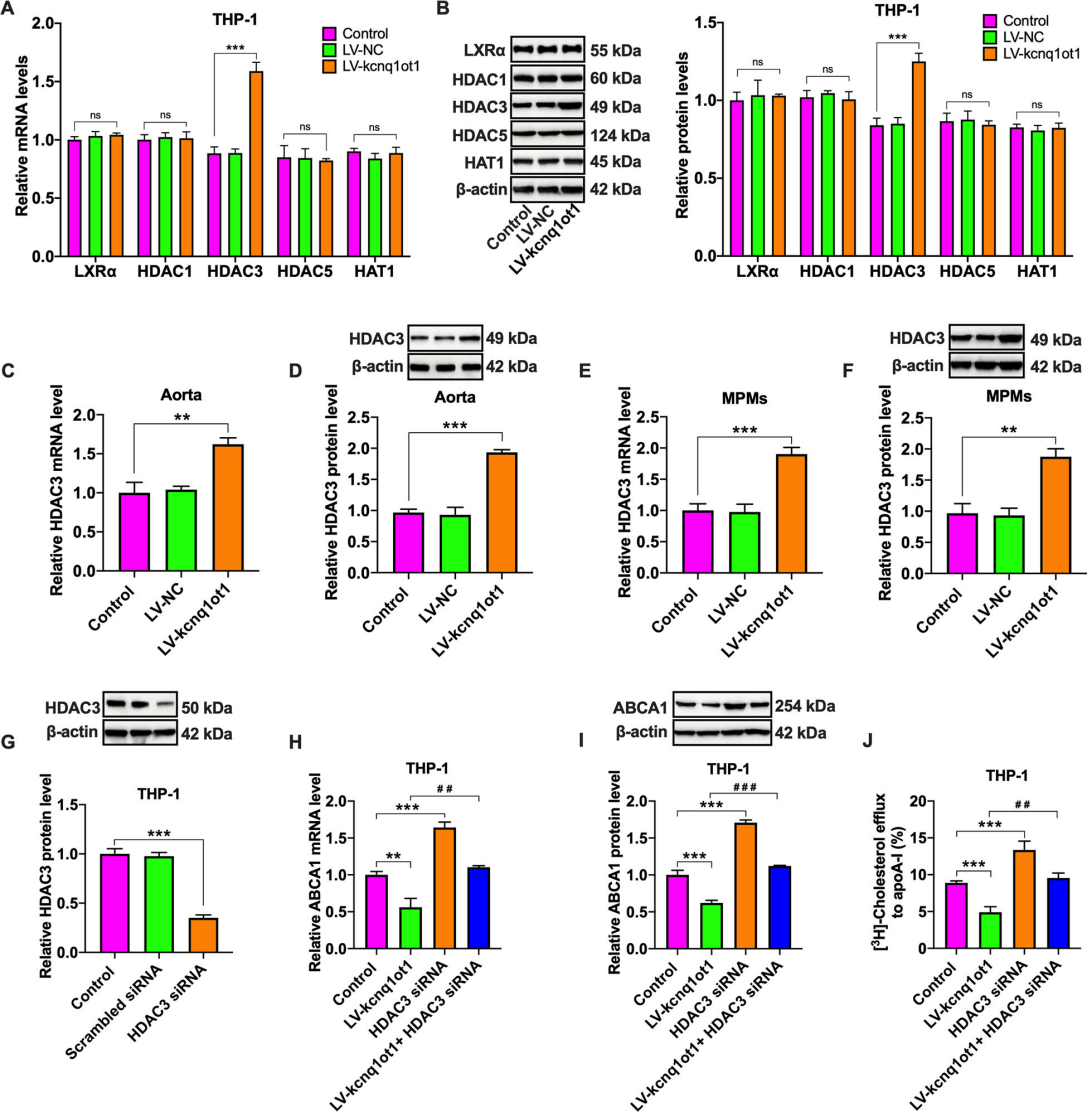
Involvement of HDAC3 in kcnq1ot1-induced downregulation of ABCA1 expression. **A**, **B** THP-1 macrophages were transfected with PBS, LV-NC or LV-kcnq1ot1 for 72 h. The expressions of LXRα, HDAC1, HDAC3, HDAC5, and HAT1 were determined by qRT-PCR and western blot (*n* = 3). **C**, **D** Evaluation of HDAC3 expression in the aorta from apoE^−/−^ mice (*n* = 10). **E**, **F** Detection of HDAC3 expression in MPMs from apoE^−/−^ mice. **G** THP-1 macrophages were transfected with scrambled siRNA or HDAC3 siRNA for 24 h, and the cell lysates were immunoblotted with indicated antibodies (*n* = 3). **H**–**J** THP-1 macrophages were transfected with HDAC3 siRNA for 24 h, followed by treatment with LV-kcnq1ot1 for another 72 h (*n* = 3). **H**, **I** The mRNA and protein levels of ABCA1 were detected by qRT-PCR and western blot, respectively. **J** Detection of cholesterol efflux to apoA-I using a liquid scintillation counter. Data are represented as mean ± SD. **P < 0.01, ***P < 0.001 vs. control group; ^##^*P* < 0.01, ^###^*P* < 0.001 vs. LV-kcnq1ot1 group; ns not significant.

LXRα, a nuclear receptor, plays a critical role in stimulating ABCA1 transcription^26^. To reveal whether LXRα is implicated in kcnq1ot1-induced downregulation of ABCA1 expression, qRT-PCR and western blot were employed to measure LXRα expression. Unexpectedly, the mRNA and protein levels of LXRα were unchangeable in THP-1 macrophages treated with LV-kcnq1ot1 (Fig. 5A, B), suggesting that kcnq1ot1 inhibits ABCA1 expression in an LXRα-independent manner.

Several lines of evidence have demonstrated that histone acetylation/deacetylation participates in the regulation of ABCA1 expression^27,28^. We inferred that the inhibitory effect of kcnq1ot1 on ABCA1 expression is likely mediated by HATs or HDACs. Our results showed that transfection of THP-1 macrophages with LV-kcnq1ot1 dramatically elevated the mRNA and protein levels of HDAC3 but had no impact on HDAC1, HDAC5, and HAT-1 expressions (Fig. 5A, B). Similar to these findings, HDAC3 expression was significantly increased in the aorta and MMPs from kcnq1ot1-overexpressing apoE^−/−^ mice (Fig. 5C–F). To address the role of HDAC3 in kcnq1ot1-induced downregulation of ABCA1 expression, THP-1 macrophages were transfected with HDAC3 siRNA. The western blot results confirmed efficient knockdown of HDAC3 expression by HDAC3 siRNA (Fig. 5G). Subsequently, THP-1 macrophages were treated with HDAC3 siRNA, followed by transfection with LV-kcnq1ot1. The inhibitory effects of kcnq1ot1 on ABCA1 expression and cholesterol efflux were completely reversed by pretreatment with HDAC3 siRNA (Fig. 5H–J). To summarize, kcnq1ot1 inhibits ABCA1 expression and cholesterol efflux via HDAC3 upregulation.

### HDAC3 is a direct target of miR-452-3p

**Fig. 6:**
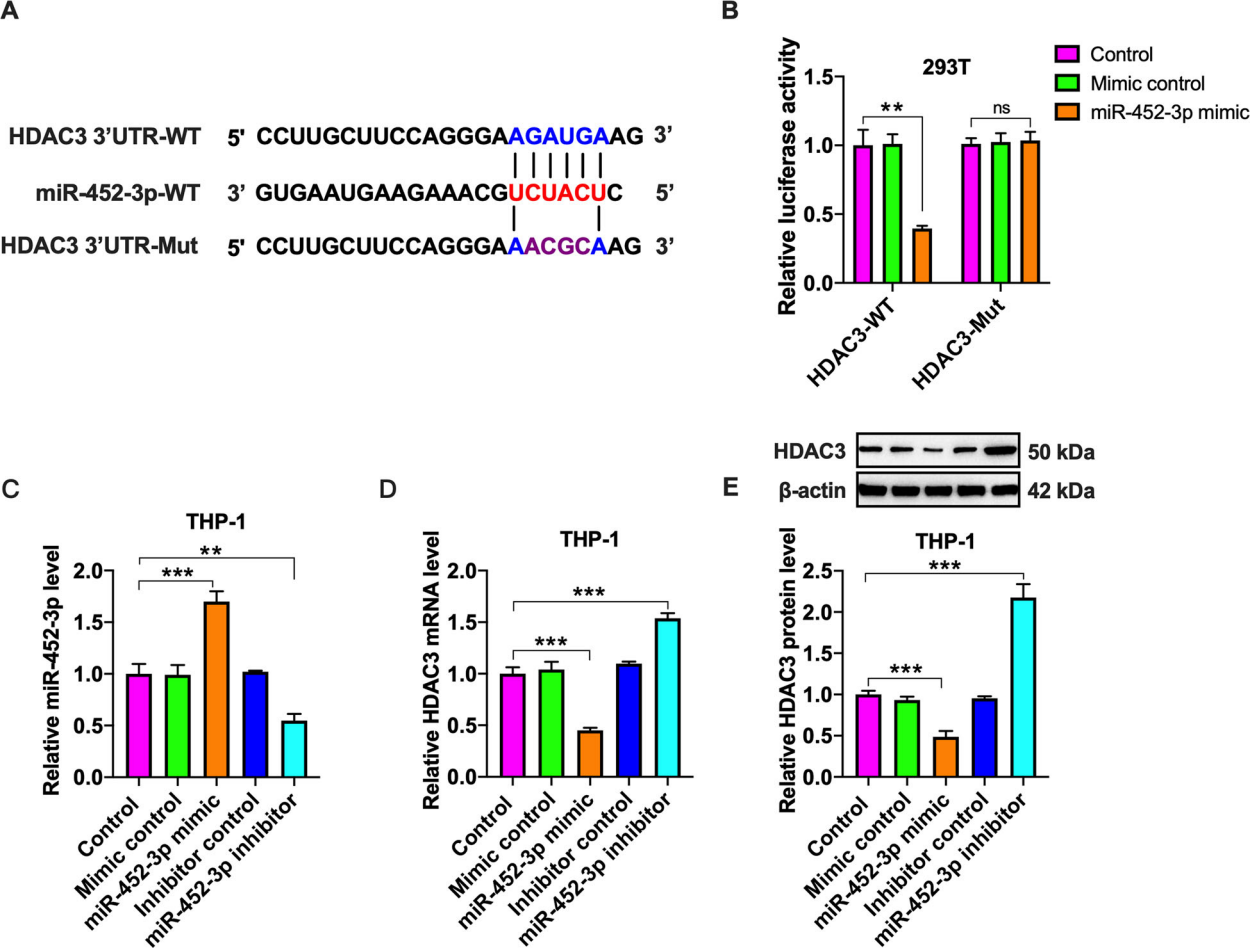
Identification of HDAC3 as a direct target of miR-452-3p. **A** Schematic of miR-452-3p binding site in the 3′UTR of HDAC3 mRNA and corresponding mutation. **B** The luciferase reporter plasmids (HDAC3-WT and HDAC3-Mut) were co-transfected into 293T cells with miR-452-3p mimic or its negative control. Luciferase activity was measured after 48 h. **C**–**E** THP-1 macrophages were transfected with mimic/inhibitor or their negative controls for 48 h; **C** qRT-PCR analysis of miR-452-3p expression. **D**, **E** The mRNA and protein levels of HDAC3 were determined by qRT-PCR and western blot, respectively. Data are represented as mean ± SD from three independent experiments. **P < 0.01, ***P < 0.001; ns not significant.

There is increasing evidence that HDAC3 is regulated by miRNAs at the transcriptional level^29,30^. To search for potential miRNAs that can target HDAC3, we performed bioinformatics analyses (miRDB and Targetscan) and found that there was a putative binding site between miR-452-3p and HDAC3 3′UTR (Fig. 6A). We then constructed a luciferase reporter plasmid containing wild-type (HDAC3-WT) or mutant (HDAC3-Mut) miR-452-3p binding site (Fig. 6A). These plasmids were transfected into 293T cells together with miR-452-3p mimic or mimic control for luciferase reporter assay. Co-transfection of HDAC3-WT and miR-452-3p mimic led to a significant decrease in the luciferase activity, whereas this effect disappeared when miR-452-3p binding site was mutated (Fig. 6B). To confirm whether miR-452-3p can directly regulate HDAC3 expression, we transfected THP-1 macrophages with miR-452-3p mimic or inhibitor and found that miR-452-3p mimic markedly decreased, whereas its inhibitor increased, miR-452-3p levels, showing a high transfection efficacy (Fig. 6C). Importantly, transfection with miR-452-3p mimic down-regulated the mRNA and protein expression of HDAC3, while an opposite effect appeared in response to miR-452-3p inhibitor (Fig. 6D, E). These bioinformatics analyses and experimental results identify HDAC3 as a direct target of miR-452-3p.

### Kcnq1ot1 acts as a ceRNA for miR-452-3p

**Fig. 7:**
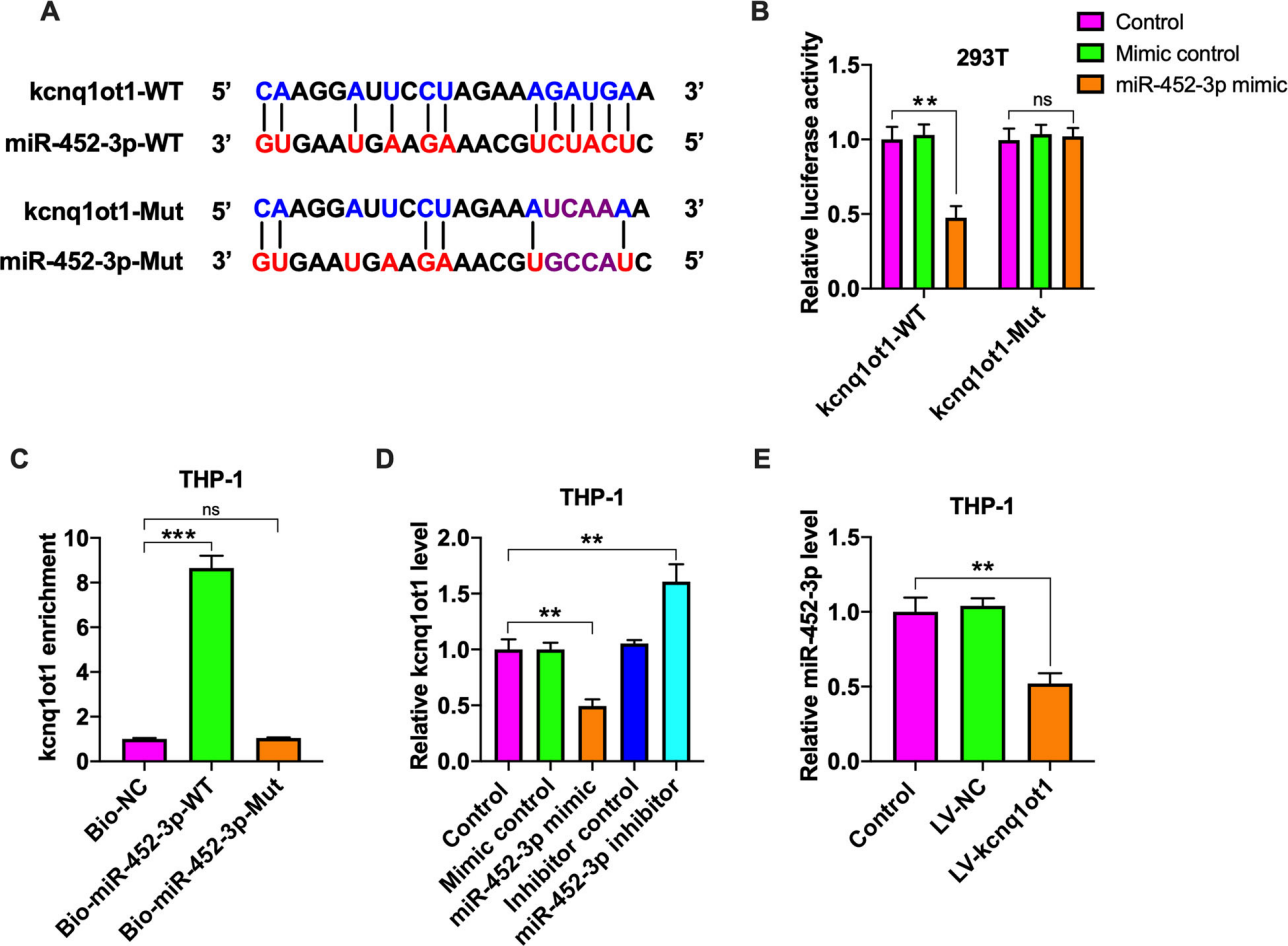
Validation of kcnq1ot1 as a ceRNA for miR-452-3p. **A** Schematic representation of miR-452-3p binding site in the kcnq1ot1 sequence and corresponding mutation. **B** The luciferase reporter plasmids kcnq1ot1-WT or kcnq1ot1-Mut were co-transfected into 293T cells with miR-452-3p mimic or its negative control for 48 h, followed by detection of luciferase activity. **C** THP-1 macrophages were transfected with bio-NC, bio-miR-452-3p-WT or bio-miR-452-3p-Mut for 48 h, which was followed by pull-down assay. **D** THP-1 macrophages were incubated with miR-452-3p mimic/inhibitor or their negative controls for 48 h. The expression of kcnq1ot1 was evaluated by qRT-PCR. **E** Following transduction of THP-1 macrophages with PBS, LV-NC, or LV-kcnq1ot1 for 72 h, qRT-PCR was conducted to detect miR-452-3p expression. Data are represented as mean ± SD from three independent experiments. **P < 0.01, ***P < 0.001; ns not significant.

In addition to protein-coding genes, miRNAs can target lncRNAs^31^. Among candidate targets of miR-452-3p predicted by the starBase v2.0 database, we focused on kcnq1ot1 because it shared a common miR-452-3p binding site with HDAC3 (Fig. 7A). The luciferase reporter assay indicated that luciferase activity of kcnq1ot1-WT was remarkably reduced by miR-452-3p mimic transfection, while luciferase activity of kcnq1ot1-Mut was not changeable (Fig. 7B). To further validate the interaction between miR-452-3p and kcnq1ot1, we employed a bio-miR-452-3p pulldown assay. Our results showed that only bio-miR-452-3p-WT could pull down kcnq1ot1 (Fig. 7C), indicating a direct interaction between the two RNAs. Next, we tested the effect of miR-452-3p on kcnq1ot1 expression in THP-1 macrophages by qRT-PCR. Transfection with miR-452-3p mimic led to a significant decrease in kcnq1ot1 levels, and miR-452-3p inhibitor had an opposite effect (Fig. 7D). At the same time, treatment of THP-1 macrophages with LV-kcnq1ot1 attenuated miR-452-3p levels (Fig. 7E) but stimulated HDAC3 expression (Fig. 5A, B). Taken together, these findings indicate the existence of specific cross-talk between kcnq1ot1 and HDAC3 through competition for miR-452-3p binding.

### Kcnq1ot1 enhances HDAC3 expression and inhibits ABCA1-mediated cholesterol efflux by sponging miR-452-3p

**Fig. 8:**
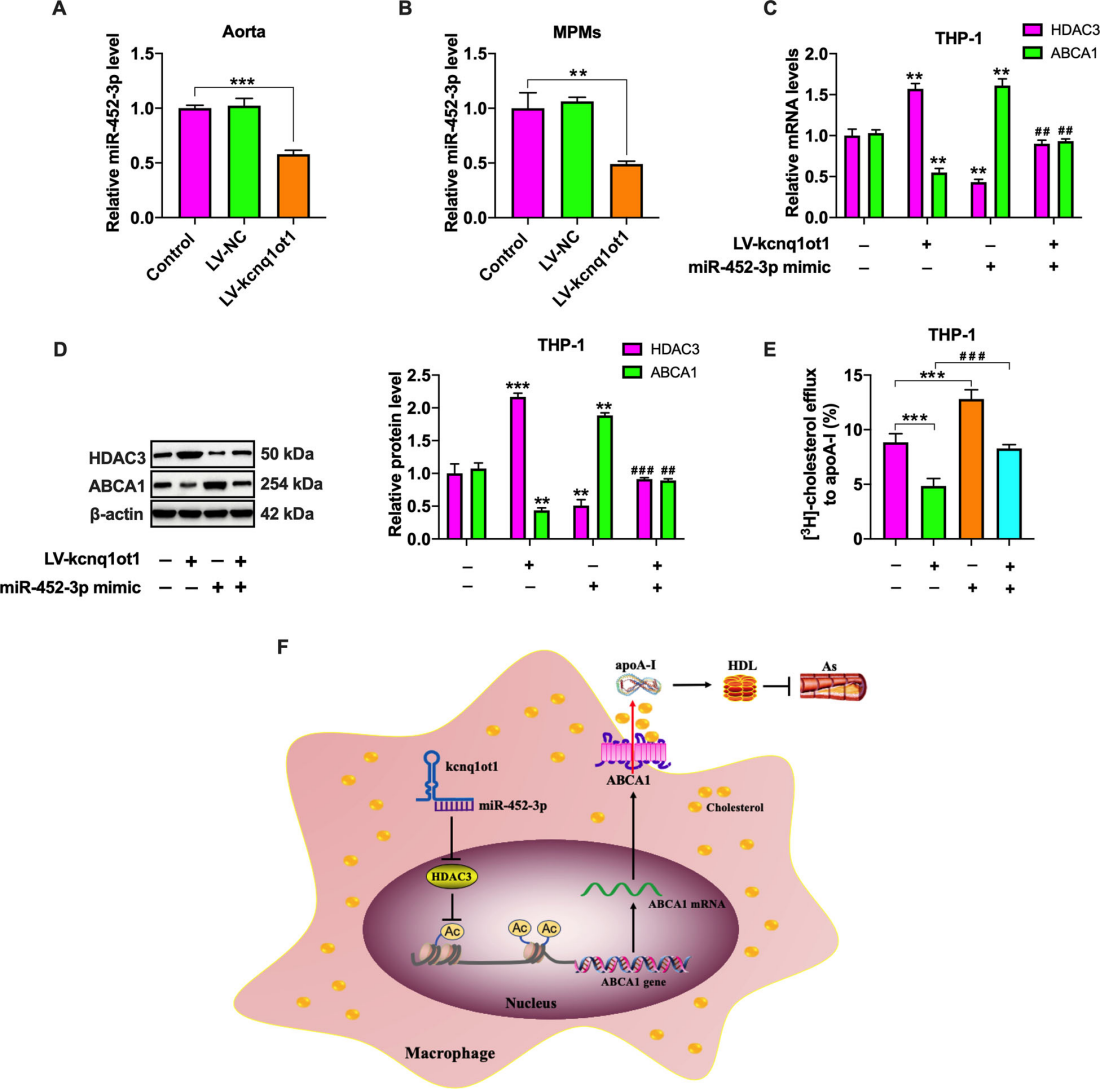
Kcnq1ot1 sponges miR-452-3p to increase HDAC3 levels and inhibit ABCA1 expression. **A**, **B** qRT-PCR analysis of miR-452-3p expression in the aorta (*n* = 10) and MPMs (*n* = 5) from apoE^−/−^ mice. **C**–**E** THP-1 macrophages were transfected with miR-452-3p mimic for 48 h and then transduced with LV-kcnq1ot1 for 72 h (*n* = 3). **C**, **D** The mRNA and protein levels of HDAC3 and ABCA1 were detected by qRT-PCR and western blot, respectively. **E** Detection of cholesterol efflux using a liquid scintillation counter. **F** Schematic overview of the proatherosclerotic action of kcnq1ot1. Kcnq1ot1 increases the levels of HDAC3 by functioning as a ceRNA for miR-452-3p, which in turn downregulates ABCA1 expression, inhibits cholesterol efflux from macrophage, reduces plasma HDL-C levels, and exacerbates atherosclerosis. Data were represented as mean ± SD. **P < 0.01, ***P < 0.001 vs. control group; ^##^*P* < 0.01, ^###^*P* < 0.001 vs. LV-kcnq1ot1 group.

Since there is a ceRNA network among kcnq1ot1, miR-452-3p and HDAC3, we wondered whether miR-452-3p is required for the regulatory effects of kcnq1ot1 on HDAC3 and ABCA1 expression. Like THP-1 macrophages, decreased miR-452-3p expression was observed in the aorta (Fig. 8A) and MPMs (Fig. 8B) of apoE^−/−^ mice treated with LV-kcnq1ot1. Subsequently, THP-1 macrophages were transfected with miR-452-3p mimic, followed by LV-kcnq1ot1 transduction. As shown in Fig. 8C–E, transfection with miR-452-3p mimic significantly abrogated the effects of kcnq1ot1 overexpression on HDAC3 and ABCA1 expressions as well as cholesterol efflux. Thus, we concluded that kcnq1ot1 increases the levels of HDAC3 by functioning as a ceRNA for miR-452-3p, thereby inhibiting ABCA1 expression and subsequent cholesterol efflux.

## Discussion

Atherosclerotic cardiovascular disease is the leading cause of death in developed countries^32,33^. There is accumulating evidence that lncRNAs are involved in the regulation of lipid metabolism and atherosclerosis^34^. Recent studies have demonstrated that kcnq1ot1 plays an important causative role in cardiovascular disease, such as cardiomyopathy and myocardial ischemia/reperfusion injury. However, until now little is known regarding the role of this lncRNA in atherogenesis. In this study, we found that kcnq1ot1 was significantly up-regulated in mouse aorta with atherosclerosis and lipid-loaded macrophages, and that kcnq1ot1 overexpression increased atherosclerotic lesion size, promoted lipid deposition, and decreased collagen content in apoE^−/−^ mice, while an opposite effect appeared in response to kcnq1ot1 knockdown. These data suggest that kcnq1ot1 contributes to atherosclerosis development.

Lipid metabolism disorder plays a central role in the occurrence and development of atherosclerosis. It is estimated that a 1 mg/dL increase in plasma HDL-C levels decreases coronary heart disease risk in men by 2% and women by 3%^35^. RCT is regarded as the major mechanism by which HDL protects against atherosclerotic cardiovascular disease. Our data demonstrated that kcnq1ot1 overexpression markedly attenuated plasma HDL-C levels and impaired RCT in apoE^−/−^ mice, further supporting the proatherogenic action of kcnq1ot1.

Lipid accumulation is essential for the transformation of macrophages into foam cells, a hallmark of atherosclerotic lesions throughout all stages of this disease. Our results showed that overexpression of kcnq1ot1 increased, whereas its inhibition decreased, intracellular cholesterol amounts and lipid droplets in THP-1 macrophages, suggesting this lncRNA as an important contributor to lipid accumulation. Increased cholesterol uptake and/or decreased cholesterol efflux leads to intracellular lipid accumulation^36^. Both CD36 and SR-A are responsible for cholesterol uptake by macrophages^37^. ABCA1 and ABCG1 mediate the efflux of cholesterol to apoA-I and HDL, respectively. In fact, more than 50% of efflux is attributed to ABCA1 in cholesterol-enriched macrophages^38,39^. Mutations in the *ABCA1* gene cause Tangier disease, which is characterized by extremely low plasma HDL-C levels and premature atherosclerosis^24^. In contrast, we and others have revealed that increased ABCA1 expression inhibits lipid accumulation and protects apoE^−/−^ mice from atherosclerosis^4^. Here, we reported that kcnq1ot1 overexpression down-regulated ABCA1 expression but did not alter the levels of ABCG1, SR-A, and CD36. This suggests that kcnq1ot1 only affects cholesterol efflux. Further analysis demonstrated that the increased expression of kcnq1ot1 through lentivirus-mediated transduction reduced cholesterol efflux to apoA-I but not HDL. Thus, prevention of ABCA1-mediated cholesterol efflux is a critical mechanism by which kcnq1ot1 facilitates lipid accumulation and aggravates atherosclerosis.

HDAC3 belongs to the class I HDACs and can suppress gene transcription by deacetylating histone tails^40^. It has been reported that HDAC3 expression is significantly increased in human ruptured atherosclerotic lesions^41^ and in the aorta of apoE^−/−^ mice in close vicinity to branch openings where disturbed blood flow occurs^15^. HDAC3 inhibition decreases ABCA1 expression in mouse bone marrow-derived macrophages^42^. In LDL receptor-deficient mice fed a high cholesterol diet, macrophage HDAC3 deletion diminishes lipid content and promotes collagen deposition within the plaques, revealing HDAC3 as an atherogenic agent^15^. Our results showed that treatment with LV-kcnq1ot1 dramatically up-regulated HDAC3 expression in the aorta and macrophages. Importantly, the decreased ABCA1 levels and cholesterol efflux could be inhibited by HDAC3 silencing, indicating the involvement of HDAC3 in kcnq1ot1-induced downregulation of ABCA1 expression.

Protein-coding genes and lncRNAs can regulate each other by competitively binding to miRNAs. These lncRNAs sequester miRNAs to release their target mRNAs, which has been defined as ceRNAs. There is increasing evidence that kcnq1ot1 acts as a ceRNA to participate in the occurrence and development of malignant tumors and diabetic cardiomyopathy^43,44^. miR-452-3p is a pleiotropic miRNA involved in metabolic diseases. In this study, we observed that kcnq1ot1 and HDAC3 shares a common miR-452-3p binding site. Further, miR-452-3p decreased the levels of kcnq1ot1 and HDAC3, and kcnq1ot1 differentially regulated miR-452-3p and HDAC3 expressions. These findings reveal a direct competition for miR-452-3p binding between kcnq1ot1 and HDAC3. Recent studies have demonstrated that the ceRNA regulation network is associated with ABCA1 expression, lipid metabolism, and atherosclerosis^45–47^. Similarly, our results showed that increased HDAC3 expression and decreased ABCA1 expression and cholesterol efflux induced by kcnq1ot1 was rescued by pretreatment with miR-452-3p mimic. We conclude that kcnq1ot1 sponges miR-452-3p to raise the levels of HDAC3, leading to downregulation of ABCA1 expression and inhibition of subsequent cholesterol efflux from macrophages. Given the complexity of lncRNA action mechanisms, it is possible that kcnq1ot1 regulates ABCA1 expression and affect atherosclerosis progression by other pathways.

In summary, the present study has demonstrated an important role of kcnq1ot1 in facilitating atherosclerosis development and revealed a novel mechanism for ABCA1 regulation. Kcnq1ot1 combines with miR-452-3p and up-regulates HDAC3 expression by acting as a ceRNA, which results in decreased ABCA1 expression, less cholesterol release, and more foam cell formation (Fig. 8F). Thus, inhibiting endogenous kcnq1ot1 expression may be a promising strategy for the prevention and treatment of atherosclerotic cardiovascular disease.

## Supplementary information

Supplementary Figure Legend

Supplementary Figure 1

Supplementary Figure 2

Supplementary Table 1

Supplementary Table 2

Supplementary Table 3
