# Supplementary material for: LncRNA kcnq1ot1 promotes lipid accumulation and accelerates atherosclerosis via functioning as a ceRNA through the miR-452-3p/HDAC3/ABCA1 axis
Source: Cell Death Dis. 2020 Dec 9;11(12):1043. doi: 10.1038/s41419-020-03263-6 (PMC7723992; doi:10.1038/s41419-020-03263-6)
Supplement: Supplementary file 1 — Supplementary Figure Legend [file 41419_2020_3263_MOESM1_ESM.docx]

**Supplementary Figure Legends**

**Supplementary Figure 1.** Effects of kcnq1ot1 knockdown on atherosclerosis in apoE^-/-^ mice. Western diet-fed apoE^-/-^ mice were injected with LV-shNC or LV-shkcnq1ot1 via the tail vein (n=10 in each group). (A) The qRT-PCR analysis of kcnq1ot1 expression in the aorta; (B) Sections of the aortic root were stained with HE, Oil Red O, or Masson﻿. Lesion area and percentage was quantified using Image-Pro Plus 7.0 software﻿. Scale bar=100 μm. ﻿Data are represented as mean ± SD. ﻿****P* < 0.001.

**Supplementary Figure 2.** Effects of kcnq1ot1 knockdown on lipid accumulation in THP-1 macrophages. THP-1 macrophages were pretreated with or without 50 µg/mL ox-LDL for 48 h, and then transduced with LV-shNC, or LV-shkcnq1ot1 for 72 h (n=3). (A) kcnq1ot1 expression was detected by qRT-PCR. (B) Representative images of Oil red O staining (400×). Scale bar=20 μm. Data are represented as mean ± SD. ****P* < 0.001.
