# Supplementary figures and images for: LncRNA kcnq1ot1 promotes lipid accumulation and accelerates atherosclerosis via functioning as a ceRNA through the miR-452-3p/HDAC3/ABCA1 axis
Source: Cell Death Dis. 2020 Dec 9;11(12):1043. doi: 10.1038/s41419-020-03263-6 (PMC7723992; doi:10.1038/s41419-020-03263-6)

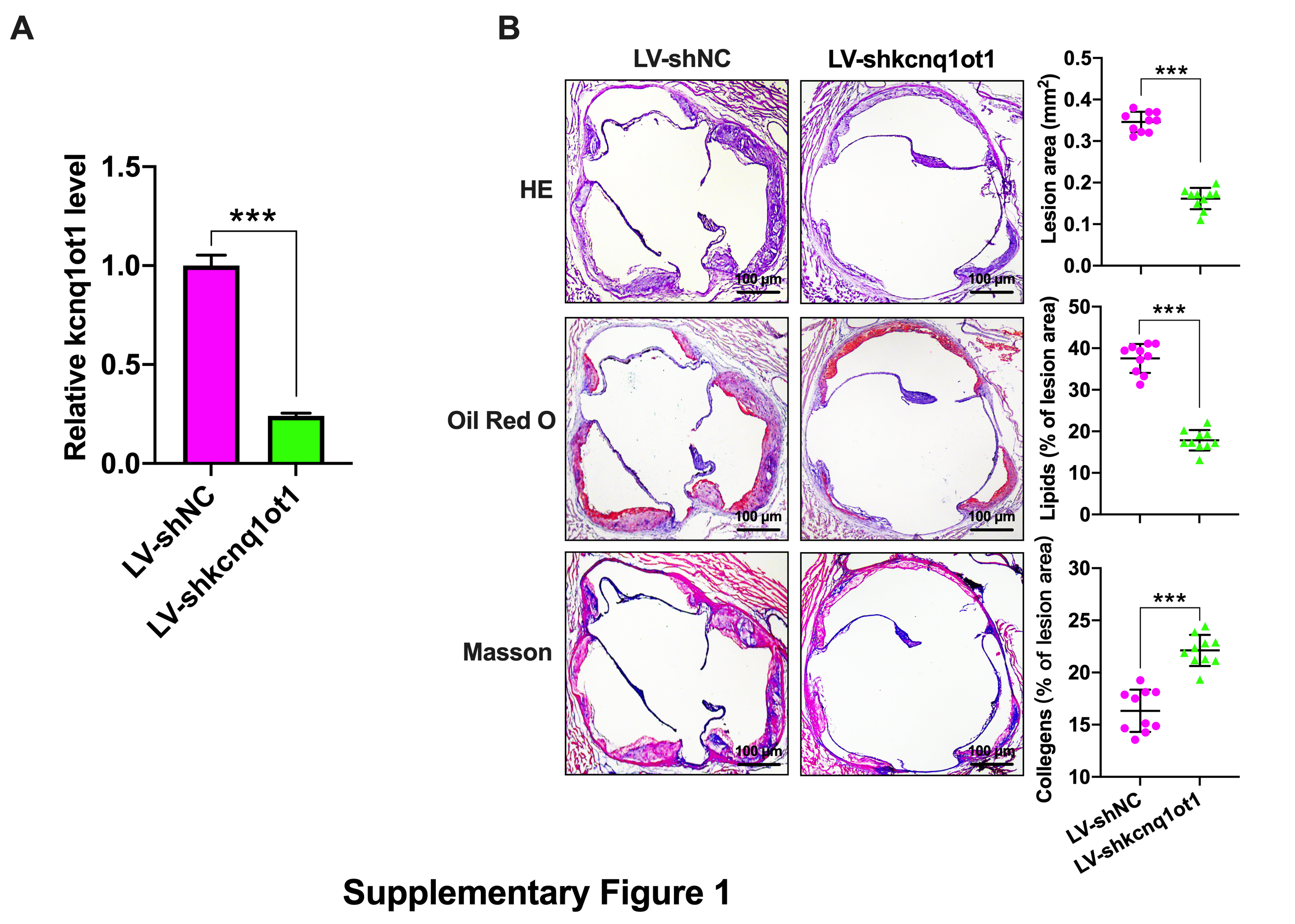

Supplement: Supplementary file 2 — Supplementary Figure 1 [file 41419_2020_3263_MOESM2_ESM.tif]

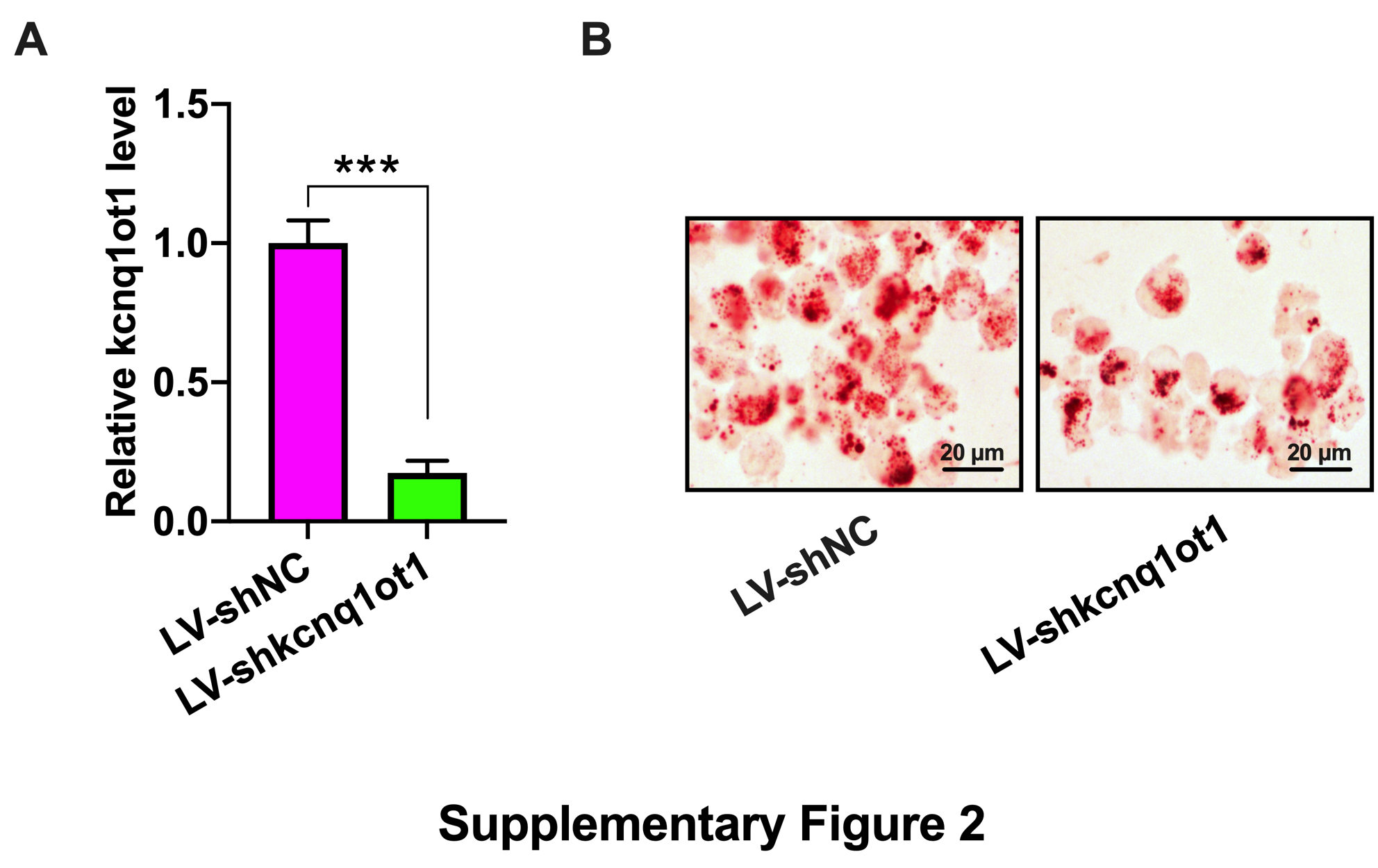

Supplement: Supplementary file 3 — Supplementary Figure 2 [file 41419_2020_3263_MOESM3_ESM.tif]
