# Supplementary material for: LncRNA kcnq1ot1 promotes lipid accumulation and accelerates atherosclerosis via functioning as a ceRNA through the miR-452-3p/HDAC3/ABCA1 axis
Source: Cell Death Dis. 2020 Dec 9;11(12):1043. doi: 10.1038/s41419-020-03263-6 (PMC7723992; doi:10.1038/s41419-020-03263-6)
Supplement: Supplementary file 4 — Supplementary Table 1 [file 41419_2020_3263_MOESM4_ESM.docx]

**Supplementary Table 1.** The primer sequences used in qRT-PCR.

| kcnq1ot1 (Human) | Forward, 5’- ACTCACTCACTCACTCACT-3’;  Reverse, 5’-CTGGCTCCTTCTATCACATT-3’ |
| --- | --- |
| kcnq1ot1 (Mouse) | Forward, 5’-GCACTCTGGGTCCTGTTCTC-3’;  Reverse, 5’-CACTTCCCTGCCTCCTACAC-3’ |
| miR-452-3p (Human) | Forward, 5’-CTCATCTGCAAAGAAGTAAGTG-3’;  Reverse, 5’-CAGTGCGTGTCGTGGAGT-3’ |
| miR-452-3p (Mouse) | Forward, 5’-TCAGTCTCATCTGCAAAGAGGT-3’;  Reverse, 5’-CAGTGCGTGTCGTGGAGT-3’ |
| ABCA1 (Human) | Forward, 5’-GTCCTCTTTCCCGATTATCTGG-3’;  Reverse, 5’-CACTCACTCTCGCTCGCAAT-3’ |
| ABCA1 (Mouse) | Forward, 5’-GGGTGGTGTTCTTCCTCATTAC-3’;  Reverse, 5’-GAATGACGAGGATGAGGATGTG-3’ |
| ABCG1 (Human) | Forward, 5’-TGTTCATCAGCGTGCACTTC-3’;  Reverse, 5’-AGGGCTCAAGCATTGTCATC-3’ |
| ABCG1 (Mouse) | Forward, 5’-AGGTCTCAGCCTTCTAAAGTTCCTC-3’;  Reverse, 5’-TCTCTCGAAGTGAATGAAATTTATCG-3’ |
| SR-A (Human) | Forward, 5’-TTTGATGCTCGCTCAATGACA-3’;  Reverse, 5’-GCTGCCACTATTCCAATGAGAG-3’ |
| SR-A (Mouse) | Forward, 5’-TGGTCCACCTGGTGCTCC-3’;  Reverse, 5’-ACCTCCAGGGAAGCCAATTT-3’ |
| CD-36 (Human) | Forward, 5’-TGCTCATCTATACACGGTTACC-3’;  Reverse, 5’-TGCTCATCTATACACGGTTACC-3’ |
| CD-36 (Mouse) | Forward, 5’-CCCAGATGCACCATGGGCTTGGCAA-3’;  Reverse, ﻿5’-AAGCTCGTGCGGCCCAGGTACT-3’ |
| LXRα (Human) | Forward, 5’-CGATCGAGGTGATGCTTCTG-3’;  Reverse, 5’-GGCAAAGTCTTCCCGGTTAT-3’ |
| HDAC1 (Human) | Forward, 5’-GCTCCACATCAGTCCTTCC-3’;  Reverse, 5’-GGTCGTCTTCGTCCTCATC-3’ |
| HDAC3 (Human) | Forward, 5’-ACCAATATGCAAGGCTTCACCAA-3’;  Reverse, 5’-GCCTGTGTAACGCGAGCAGA-3’ |
| HDAC3 (Mouse) | Forward, 5’-CGAGGAGAACTACAGCAGG-3’;  Reverse, 5’-AGAGGGACAATCATCAGG-3’ |
| HDAC5 (Human) | Forward, 5’-TCCCTCCTACAAATTGCC-3’;  Reverse, 5’-GGTGATCTCAACTGCTCTC-3’ |
| HAT-1 (Human) | Forward, 5’-GGATGGAGCTACGCTCTTTG-3’;  Reverse, 5’-GGATGGATCTTCCGCTGTAA-3’ |
| β-actin (Human) | Forward, 5’-GACCTCTATGCCAACACAGT-3’;  Reverse, 5’-AGTACTTGCGCTCAGGAGGA-3’ |
| β-actin (Mouse) | Forward, 5’-TGGCACCCAGCACAATGAA-3’;  Reverse, 5’-CTAAGTCATAGTCCGCCTAGAAGCA-3’ |
| U6 (Human) | Forward, 5’-GCTTCGGCAGCACATATACTAAAAT-3’;  Reverse, 5’-CGCTTCACGAATTTGCGTGTCAT-3’ |
| U6 (Mouse) | Forward, 5’-CTCGCTTCGGCAGCACATATACT-3’;  Reverse, 5’-ACGCTTCACGAATTTGCGTGTC-3’ |
