# Supplementary material for: LncRNA kcnq1ot1 promotes lipid accumulation and accelerates atherosclerosis via functioning as a ceRNA through the miR-452-3p/HDAC3/ABCA1 axis
Source: Cell Death Dis. 2020 Dec 9;11(12):1043. doi: 10.1038/s41419-020-03263-6 (PMC7723992; doi:10.1038/s41419-020-03263-6)
Supplement: Supplementary file 5 — Supplementary Table 2 [file 41419_2020_3263_MOESM5_ESM.docx]

**Supplementary Table 2.** Effects of kcnq1ot1 on intracellular cholesterol contents in THP-1 macrophages.

| Group | TC (μg/mg) | FC (μg/mg) | CE (μg/mg) | CE/TC (%) |
| --- | --- | --- | --- | --- |
| Control | 485.2±23.1 | 168.7±15.6 | 316.5±19.8 | 65.2 |
| LV-NC | 477.3±33.2 | 158.9±25.5 | 318.4±18.7 | 66.7 |
| LV-kcnq1ot1 | 548.9±21.2* | 187.1±23.1* | 361.8±22.8* | 65.9 |

**P* < 0.05 *vs.* control group.
