# Supplementary material for: LncRNA kcnq1ot1 promotes lipid accumulation and accelerates atherosclerosis via functioning as a ceRNA through the miR-452-3p/HDAC3/ABCA1 axis
Source: Cell Death Dis. 2020 Dec 9;11(12):1043. doi: 10.1038/s41419-020-03263-6 (PMC7723992; doi:10.1038/s41419-020-03263-6)
Supplement: Supplementary file 6 — Supplementary Table 3 [file 41419_2020_3263_MOESM6_ESM.docx]

**Supplementary Table 3.** Effects of kcnq1ot1 knockdown on intracellular cholesterol contents in THP-1 macrophages.

| Group | TC (μg/mg) | FC (μg/mg) | CE (μg/mg) | CE/TC (%) |
| --- | --- | --- | --- | --- |
| LV-shNC | 551.4±21.7 | 221.7±23.5 | 329.7±15.2 | 59.8 |
| LV-shkcnq1ot1 | 409.8±13.1* | 167.3±14.2* | 242.5±18.1* | 59.2 |

**P* < 0.05 *vs.* control group.
